# *Trichomonas vaginalis* Incidence Associated with Hormonal Contraceptive Use and HIV Infection among Women in Rakai, Uganda

**DOI:** 10.1155/2014/916597

**Published:** 2014-03-04

**Authors:** Heena Brahmbhatt, Richard Musoke, Frederick Makumbi, Godfrey Kigozi, David Serwadda, Maria Wawer, Ronald Gray

**Affiliations:** ^1^Department of Population, Reproductive and Family Health, Johns Hopkins Bloomberg School of Public Health, 615 North Wolfe Street, E4010, Baltimore, MD 21205, USA; ^2^Rakai Health Science Program, P.O. Box 279, Kalisizo, Uganda; ^3^Department of Statistics and Epidemiology, Makerere University, School of Public Health, New Mulago Hospital Complex, Maksph Building, P.O. Box 7072, Kampala, Uganda; ^4^Department of Epidemiology, Johns Hopkins Bloomberg School of Public Health, 615 North Wolfe Street, Baltimore, MD 21205, USA

## Abstract

*Background*. Data on the incidence of *Trichomonas vaginalis* and use of hormonal contraception (HC) are limited. *Methods*. 2,374 sexually active women aged 15–49 years from cohort surveys in Rakai, Uganda, were included. Incidence of *T. vaginalis* was estimated per 100 person years (py) and association between HC (DMPA, Norplant, and oral contraceptives) and* T. vaginalis* infection was assessed by incidence rate ratios (IRR), using Poisson regression models. *Results*. At baseline, 34.9% had used HC in the last 12 months, 12.8% HIV+, 39.7% with high BV-scores (7–10), and 3.1% syphilis positive. The 12-month incidence of *T. vaginalis *was 2.4/100 py; CI (1.90, 3.25). When stratified by type of HC used, compared to women who did not use HC or condoms, incidence of *T. vaginalis* was significantly higher among users of Norplant (adj.IRR = 3.01, CI: 1.07–8.49) and significantly lower among DMPA users (adj.IRR = 0.55, CI: 0.30, 0.98) and women who discontinued HC use at follow-up (adj.IRR = 0.30, CI: 0.09, 0.99). HIV infection was associated with an increase in incidence of *T. vaginalis* (adj.IRR = 2.34, CI: 1.44, 3.78). *Conclusions*. Use of Norplant and being HIV+ significantly increased the risk of *T. vaginalis*, while use of DMPA and discontinuation of overall HC use were associated with a decreased incidence of *T. vaginalis.*

## 1. Introduction

*Trichomonas vaginalis* (*T. vaginalis*) is the most common curable sexually transmitted infection (STI), and despite high prevalence rates ranging from 5–75%, and adverse health consequences such as pelvic inflammatory disease and adverse pregnancy outcomes, it has received little attention globally [1]. Sub-Saharan Africa continues to bear a disproportionate burden of the HIV disease with an estimated 22.5 million people living with HIV [2] and *T. vaginalis* has been shown to increase the odds of HIV acquisition between 1.5-fold and 3-fold [3, 4]. Dual use of efficacious contraceptives and condoms continues to be encouraged to prevent unwanted pregnancies and the high rates of HIV and STIs; however, dual use continues to be uncommon, especially among stable unions.

The use of hormonal contraception (HC) and especially injectable contraceptives (ICs) is gaining popularity in Sub-Saharan Africa [5], and the two latest Ugandan demographic and health survey (DHS) show an increase in use of HCs among currently married women aged 15 to 49 years from 13.4% in 2005/6 [6] to 19.7% in 2011 [7]. Given that *T. vaginalis* is highly asymptomatic in females and their male partners and rates of reinfection are consequently high, efforts need to focus on integration of *T. vaginalis* screening and prevention in family planning, reproductive health, and HIV care centres as syndromic management alone may not be enough.

Studies suggested that women using HC and especially progesterone-only contraception, may be at increased risk for HIV acquisition and other STIs. A systematic review found that injectable contraceptives (IC) increased risk of HIV transmission [8, 9], by some, but not all, studies. With the lack of consistent evidence regarding the association of HC and HIV infection, the current WHO guidelines recommend that women at high risk for HIV infection can use any HC, with encouragement of concomitant use of condoms [10].

A recent examination of the association of HC (Combined Oral Contraceptives-COCs and DMPA) and risk of STIs found no association between *T. vaginalis* and COCs in a prospective study among sex workers in Kenya but a reduced risk associated with Norplant [4]. Results from studies assessing impact of DMPA use and *T. vaginalis* were mixed as some studies found no association [11, 12], while others found a decreased risk [11, 13].

Of the studies that did find a decrease in the risk of *T. vaginalis* associated with DMPA, none were population based studies; one was a prospective study of sex workers in Kenya [11] and another on women attending family planning clinics in South Africa [13]. There are insufficient data about the determinants of *T. vaginalis* among women from rural community based settings. Given the popularity of HC among African women, understanding the impact of HC on STI risk is important.

The purpose of our study was to assess determinants of *T. vaginalis* and, specifically, the impact of HIV infection and HC on incidence of *T. vaginalis* among women from rural Rakai, Uganda.

## 2. Methods

Rural Rakai district located in southwest Uganda is the site of a prospective open cohort study called the Rakai Community Cohort Study (RCCS). We conducted retrospective analyses of data from two rounds of RCCS conducted in 2011 and 2012 to assess risk factors of *T. vaginalis* among consenting women aged 15 to 49 years resident in 50 communities.

Details have been described previously [14, 15], but, briefly, detailed information is collected on RCCS participants annually on sociodemographic, sexual behaviour, and contraceptive use. In addition, biological specimens are collected to test for HIV and STIs such as *T. vaginalis*, bacterial vaginosis, and syphilis.

Biological samples were collected in the home after the interview. HIV status was determined from venous blood using two different enzyme immunoassays (Vironostika HIV-1, OrganonTeknika, Charlotte, North Carolina, USA, and Cambridge Biotech, Worcester, Massachusetts, USA), with Western blot (HIV-1 WB Bio-Merieux-Vitek, St Louis, Missouri, USA) confirmation of all discordant EIAs and all HIV seroconverters. Syphilis was screened for in each round using the nontreponemal TRUST (Toluidine Red Unheated Serum Test, New Horizons, Columbia, MD, USA), with positive samples confirmed with the use of *Treponema pallidum *haemagglutination (Sera-Tek, Fujirebio, Tokyo, Japan). Two self-collected vaginal swabs were obtained from all consenting women in the cohort. One swab was inoculated into a *Trichomonas vaginalis (T. vaginalis)* culture kit (InPouch TV, BioMed Diagnostics, San Jose, CA, USA). In order to assess incidence of *T. vaginalis*, all women with complete information on *T. vaginalis *and HIV serostatus from rounds 14 (2010-11) and 15 (2011-12) were included in these analyses. The second swab was gram-stained and read for bacterial vaginosis by the quantitative, morphological Nugent scoring system.

Covariates considered in our analyses were (i) age stratified as less than 20 years, 20–29 years, 30–39 years, and 40+ years, (ii) marital status categorized as never married, currently married, or previously married and among married, women in monogamous versus polygamous unions, (iii) education classified as none, primary, and secondary or more, (iv) religion classified as Catholic/Protestant, Saved/Pentecostal, or other, (v) number of sexual partners in the past 12 months classified as 1 or 2 or more, (vi) socioeconomic status (SES) based on the dwelling construction materials with high SES if modern construction materials (iron/tiles, cement) were used, (vii) Nugent score of 0–3, 4–6, and 7–10 (BV), (viii) syphilis result, and (ix) HIV infection. Four different variables were created for contraceptive use exposure (Tables 2(a)–2(d)). (i) No HC/no condoms (reference group) and the exposure group consisted of 2 groups of women: women who used HC only and women who used condoms only; (ii) no HC/no condoms (reference group) and exposure was 4 groups of women: DMPA only, COC only, implants only, and condoms only; (iii) they consistently used no contraception method at baseline and follow-up (reference group) and exposure was 4 groups of women; they consistently used HCs only at baseline and follow-up, initiated HCs at follow-up, discontinued HCs at follow-up, and consistently used condoms only at baseline and follow-up;(iv) they consistently used no contraception method at baseline or follow-up (reference group) and exposure groups were women who consistently used DMPA only at baseline and follow-up, consistently used COCs at baseline and follow-up, and consistently used implants only at baseline and follow-up and other contraceptive use category (includes all women who were not included in the categories above for this table).

Statistical tests used were chi-squared analyses for comparison between groups defined by contraceptive exposure. The incidence of *T. vaginalis* was determined as the number of new *T. vaginalis*-positive women at the follow-up visit, estimated per 100 person-years (py). *T. vaginalis* infection was assumed to have occurred at mid-interval between surveys. The association between HC and incidence *T. vaginalis* was assessed by use of incidence rate ratios (IRR) through the Poisson regression models. Subanalyses were done to determine the effect of each HC method on T. vaginalis incidence as well as impact of continuation of use between the two rounds of follow-up. All variables in the bivariate analyses with *P* < 0.15, potential confounders, or variables with an IRR ≥2.0 or ≤0.5 were included in the adjusted model to obtain independent effects of HC use on incidence *T. vaginalis*. All variables in the multivariate model were assessed for collinearity using the variance inflation factor (VIF) and variables found to be highly correlated were excluded from the multivariate analyses. Covariates in all the analyses included age, marital status, education, social-economic status (SES), condom use, and other STIs. The interaction between HC use and HIV status was conducted and tested for statistical significance using the Wald-test. Model goodness of fit was tested using log-likelihood tests. All statistical analyses were conducted using STATA software package version 12.0 (College Station, Texas, USA).

The studies were approved by Institutional Review Boards in Uganda (the Scientific and Ethics Committee of the Uganda Virus Research Institute and the Uganda National Council of Science and Technology) and the United States (Western IRB, Olympia, Washington, and the Johns Hopkins University, School of Public Health).

## 3. Results

Overall, 2,374 sexually active, consenting women aged 15 to 49 years with complete data on *T. vaginalis* and HIV infection status between round 14 (2010-2011) and round 15 (2011-2012) were included in these analyses. All women in this analyses tested negative for* T. vaginalis* at baseline.

The primary outcome of interest was incidence of *T. vaginalis* associated with hormonal contraceptive (HC) use. The analyses assessed risk of *T. vaginalis* associated with overall hormonal contraception (HC) use as well as stratified by type of HC used: Depo-Provera (DMPA), combined oral contraceptives (COCs), and implants (Norplant). All adjusted analyses controlled for potential confounders such as infection with other STIs such as bacterial vaginosis (BV) and syphilis, HIV infection, and number of sexual partners in the past 12 months. In addition, the risk for *T. vaginalis* was assessed by consistency and continuation of HC use between the two rounds of follow-up.

In this analysis, which excludes other modern contraceptive methods, the overall 12 months prevalence of use of HC, was 34.9% (829/2374). Among HC users, the most commonly used types were DMPA (81.3%), COCs (15.1%), or Norplant (3.7%). The proportion of women who tested positive for *T. vaginalis* at the end of the 12-month follow-up visit was 4.0% (96/2374), resulting in an incidence of 2.4/100 py (1.90, 3.25). A total of 206 *T. vaginalis*-positive women at baseline were excluded from the analyses as our goal was to examine incidence of *T. vaginalis* associated with HC use. These initially *T. vaginalis*-negative women had lower risk characteristics than women with baseline prevalent *T. vaginalis*. For example, the proportions with 2 or more sex partners were 1.4% among the baseline *T. vaginalis*-negative compared with 5.8% among the baseline *T. vaginalis*-positive participants (*P* < 0.01).

Table 1 provides a summary of baseline characteristics by contraceptive use stratified by contraceptive use: no HC or condom use, HC use (DMPA, COCs, Norplant), and condom use alone. A significantly higher proportion of HC users are between 20 and 39 years of age, whereas a higher proportion of individuals less than 20 years were condom users. Condom users were also significantly more likely to be never or previously married and HC users were significantly more likely to be currently married. Condom users were significantly less likely to have primary or higher education, and HC and condom users were significantly more likely to be classified as high socio-economic status (SES), compared to women not using HC or using condoms alone. When looking at multiple sexual partners by contraceptive use, HC users were significantly less likely to have 2 or more partners compared to women who used condoms alone or women who used neither HC nor condoms. When looking at HIV infection rates by contraceptive use, a significantly higher proportion of condom users were HIV-positive compared to women who used HC or did not use HC or condoms. There was no significant difference by religion, BV-scores, or syphilis infection by contraceptive use.

Overall, 12.8% of the women were HIV-positive 39.7% with high BV-scores (7–10) and 3.4% syphilis-positive at baseline. *T. vaginalis* incidence was significantly higher among HIV-positive (5.0/100 py) versus HIV-negative women (2.0/100 py, *P* < 0.0001). There was no significant difference in HIV incidence rates of women who tested positive versus negative for *T. vaginalis* at follow-up (0.46/100 py and 0.50/100 py, resp.; *P* = 1.00).

Tables 2(a)–2(d) summarize *T. vaginalis* incidence by demographic and behavioural characteristics.  Table 2(a) summarizes *T. vaginalis* incidence by HC or condom use. After adjusting for sociodemographic and behavioral risk factors, syphilis infection, and BV status, factors associated with an increased risk of incident *T. vaginalis *were HIV-positive (adj.IRR = 2.34, CI: 1.44, 3.78), whereas factors that decreased the risk of *T. vaginalis* were over 40 years and were having secondary or more education.  Table 2(b) summarizes *T. vaginalis* incidence by type of contraceptive method used. In the adjusted model, factors associated with an increased risk of incident *T. vaginalis* were using implants only (adj.IRR = 3.01, CI: 1.07, 8.49) and were HIV-positive (adj.IRR = 2.38, CI: 1.47, 3.86), and factors that decreased the risk of *T. vaginalis* were using DMPA only (adj.IRR = 0.54, CI: 0.30, 0.98), being over 40 years and having secondary or more education.  Table 2(c) summarizes *T. vaginalis* incidence by consistency of contraceptive use at baseline and follow-up. In the adjusted model, factors associated with an increased risk of Incident *T. vaginalis* were HIV-positive (adj.IRR = 2.36, CI: 1.44, 3.85), whereas factors associated with a decreased risk of *T. vaginalis* were discontinuing HC use at follow-up (adj.IRR = 0.30, CI: 0.09, 0.99), being over 40 years and having secondary or higher education.  Table 2(d) summarizes *T. vaginalis* incidence by consistency of HC use at baseline and follow-up. In the adjusted model, factors associated with an increased risk of incident *T. vaginalis* were consistently using implants only (adj.IRR = 3.13, CI: 1.08, 9.07), having 1 sexual partner compared to none (adj.IRR = 1.61, CI: 1.01, 2.55) and being HIV infected (adj.IRR = 2.52, CI: 1.56, 4.08), whereas factors associated with a decrease in the risk of *T. vaginalis* were 40 years or older and were having secondary or higher education.

When assessing the impact of HC on *T. vaginalis* incidence (Tables 2(a)–2(d)), factors that were associated with an increased risk of *T. vaginalis* in the unadjusted analyses were use of Norplant (IRR = 2.75, CI: 1.00–7.65), having one versus no sexual partner in the past 12 months (IRR = 1.66, CI: 1.07, 2.56) and being HIV seropositive (IRR = 2.40, CI: 1.42, 4.08). Factors associated with a decrease in the incidence of *T. vaginalis* were using DMPA only in the past 12 months (IRR = 0.53, CI: 0.30–0.95), discontinuing HC during the follow-up period (IRR = 0.30, CI: 0.09–1.00), being over 40 years of age and having a secondary or higher education.

After adjusting for sociodemographic, behavioral risk factors, syphilis infection, and BV status, factors associated with increased risk of incident *T. vaginalis* infection were use of Norplant in the past 12 months (adj.IRR = 2.82, CI: 1.00, 8.0) and being HIV seropositive (adj.IRR = 2.45, CI: 1.51, 3.97). Factors associated with a decrease in the risk of *T. vaginalis* were use of DMPA in the past 12 months (adj.IRR = 0.53, CI: 0.29, 0.97), discontinuing HC in the past 12 months (IRR = 0.30, CI: 0.09–0.99), being over 40 years of age and having secondary education or higher.

Considering HC use at baseline and follow-up, the risk of *T. vaginalis* increased with consistent use of COCs (adj.IRR = 1.15, CI: 0.27, 4.97) and Norplant (adj.IRR = 2.76, CI: 0.92, 8.30) but decreased among women who consistently used DMPA (IRR = 0.58, CI: 0.27, 1.24) as shown in Table 2.

Interactions between HIV infection and use of Norplant were not statistically significant.

## 4. Discussion

In this longitudinal study of women in rural Uganda, use of implants (Norplant) and being HIV seropositive were associated with significantly increased incidence of *T. vaginalis*, and use of DMPA and discontinuation of hormonal contraception significantly decreased the incidence of *T. vaginalis* after controlling for HIV risk behaviors and other sexually transmitted infections (STIs) such as syphilis and bacterial vaginosis.

A number of studies have shown an increase in HIV infection associated with *T. vaginalis* [3, 4, 16]. Our study found similar rates of HIV incidence among women who tested positive for *T. vaginalis* at follow-up versus those who did not. However, power may be limited by the small number of seroconverters in the follow-up period. There are relatively few studies examining the effects of HIV on STI transmission and most are cross-sectional [17]. Although studies have not found an increase in the prevalence, incidence, persistence, or recurrence of *T. vaginalis* among women who are HIV-positive [18], our study found a significantly higher incidence of *T. vaginalis* among women who were HIV-positive, but the possibility of confounding due to correlated sexual behaviours cannot be excluded.

A recent review examined the association of hormonal contraception (COCs and DMPA) and risk of STIs and found that several longitudinal studies demonstrated increased risk of STIs such as chlamydia and gonorrhoea associated with DMPA and COCs [11, 19, 20]. The association between incidence of *T. vaginalis* and hormonal contraceptives (HC) was examined by one previous longitudinal study that found a decrease in *T. vaginalis* incidence associated with oral contraceptives, compared to women using the IUD or tubal ligation [21]. This study is the only one to date which has found an increase in incidence of *T. vaginalis* associated with the use of Norplant. These results are of concern given that the most recent Ugandan DHS surveys (2004/5 [6] and 2011 [7]) showed an 89% increase in the women reporting use of Norplant. Although the number of women using Norplant in our study was small, the increased Norplant use in Uganda and the potential risk associated with an increased risk of *T. vaginalis* warranted further investigation.

Studies have found that both endogenous hormones based on stage of menstrual cycle and use of HCs are associated with an increased susceptibility to STIs. Although the molecular and cellular mechanisms by which HC increases HIV/STI acquisition are not well understood, HC is believed to increase risk through alteration of immune responses [11, 22], structural changes of the genital tract [23], and alterations in genital flora and increased acquisition of STIs [11, 19, 20]. Infection with *T. vaginalis* has been shown to result in increased interactions of human cervicovaginal epithelial cells to the surface lipophosphoglycan of the parasite *T. vaginalis* which results in upregulation of cytokines and inflammatory processes in the genital tract microflora which may increase susceptibility to BV as well as HIV infection [24]. The reduction in the risk of *T. vaginalis* associated with DMPA found in this and other studies [11, 13] is contrary to the effect seen on other STIs. Some of the reasons hypothesized for this effect are that *T. vaginalis* possesses both specific estrogen and androgen receptors, and the androgen receptor is most closely related to the progesterone receptor. Progestins found in higher dosages in DMPA can block the androgen receptor and prevent infection of *T. vaginalis* [25].

The levels of unmet need for spacing and limiting fertility among Ugandan women remain high (34%) [7] and to achieve dual protection against STIs and HIV infection, as well as an unwanted pregnancy, couples need to use reliable contraceptives which include condoms. However, in reality, dual use is uncommon. Demographic and health surveys from 14 countries in Sub-Saharan Africa found that only 6% of youth who were currently using a condom during their last sexual act were also using a nonbarrier form of contraception [26], and dual use among couples in our setting was only reported by 3.7% of females and 8.3% of males. Efforts to control the spread of *T. vaginalis* need to focus on efforts to improve screening and identification of people at risk of *T. vaginalis* and how the epidemiology of this STI is modified by HIV infection.

Some of the limitations of this study were that this was a cohort study where assessments were done annually and hence we were not able to measure changes in contraceptive use as well as TV infection during the course of the past 12 months and this could have led to possible misclassification bias for exposure of contraceptive use. However, we have tried to assess contraceptive use at baseline and follow-up and consistency of contraceptive use at both rounds of follow-up to try and minimize any potential misclassification.

## 5. Conclusions

*Trichomonas vaginalis* incidence is higher among women using Norplant in the past 12 months, and use of DMPA or discontinuation of hormonal contraception decreased the incidence of *Trichomonas vaginalis.*

Modification of the risk of *T. vaginalis* associated with the use of some hormonal contraceptives needs to be examined further, specifically the increase in risk associated with the use of Norplant. Studies are needed to better understand the etiology and transmission of *T. vaginalis* and strategies to control *T. vaginalis* need to focus on integration of reproductive health, STI control, and HIV care services.

## Figures and Tables

**Table 1:** Baseline and demographic characteristics.

	Total		No HC/no condom use reported		COCs/injectables/implant		Condom only		*P* value
	Number	%	Number	%	Number	%	Number	%	
Overall	2374	100	1054	100	829	100	491	100	
Age									
<20	165	7	82	7.8	18	2.2	65	13.2	<0.001
20–29	939	39.6	424	40.2	367	44.3	148	30.1	
30–39	951	40.1	373	35.4	382	46.1	196	39.9	
40+	319	13.4	175	16.6	62	7.5	82	16.7	
Marital status									
Never married	212	8.9	59	5.6	39	4.7	114	23.2	<0.001
Currently married	1887	79.5	895	84.9	728	87.8	264	53.8	
Monogamous	1500	79.3	721	80.6	575	79	204	77.3	
Polygamous	387	20.7	174	19.4	153	21	60	22.7	
Previously married	275	11.6	100	9.5	62	7.5	113	23	
Education									
None	102	4.3	58	5.5	24	2.9	20	4.1	<0.001
Primary	1426	60.1	672	63.8	488	58.9	266	54.2	
Secondary or more	846	35.6	324	30.7	317	38.2	205	41.8	
Religion									
Other	328	13.8	154	14.6	114	13.8	60	12.2	0.122
Catholic/Protestant	1977	83.3	860	81.6	697	84.1	420	85.5	
Saved/Pentecostal	69	2.9	40	3.8	18	2.2	11	2.2	
Nonmarital sexual partners in last 12 months									
0	1835	77.3	880	83.5	719	86.7	236	48.1	<0.001
1	506	21.3	167	15.8	105	12.7	234	47.7	
2+	33	1.4	7	0.7	5	0.6	21	4.3	
Social-economic status									
High	1311	55.2	518	49.1	502	60.6	291	59.3	<0.001
Middle	722	30.4	352	33.4	226	27.3	144	29.3	
Low	341	14.4	184	17.5	101	12.2	56	11.4	
HIV status									
Negative	2069	87.2	961	91.2	752	90.7	356	71.5	<0.001
Positive	305	12.8	93	8.8	77	9.3	135	28.5	
BV-scores									
0–3	1093	46	475	45.1	386	46.6	232	47.3	0.316
4–6	339	14.3	145	13.8	132	15.9	62	12.6	
7–10	942	39.7	434	41.2	311	37.5	197	40.1	
Syphilis result									
Negative	2300	96.9	1020	96.8	805	97.1	475	96.7	0.901
Positive	74	3.1	34	3.2	24	2.9	16	3.3	

**(a):** 

	*N*	Incident *T. vaginalis n*/PY	Incidence/100 py	Unadj.IRR 95% CI	Adj.IRR 95% CI
Overall	2374	96/3924.6	2.4		
HC use in last 12 months					
No HC/no condom	1054	45/1733.2	2.60	1.00	1.00
HC	829	24/1366.2	1.8	0.68 (0.41–1.11)	0.71 (0.43–1.17)
Condoms only	491	27/825.2	3.3	1.3 (0.78–2.03)	0.98 (0.58–1.67)
Age					
<20	165	11/277.7	4.0	1.00	1.00
20–29	939	40/1566.3	2.6	0.64 (0.33–1.26)	0.71 (0.35–1.44)
30–39	951	37/1565.8	2.4	0.60 (0.30–1.17)	0.58 (0.28–1.18)
40+	319	8/514.8	1.6	0.39 (0.16–0.98)∗	0.29 (0.11–0.74)∗
Marital status					
Currently married	1887	69/3106.7	2.2	1.00	
Previously married	275	14/459.8	3.0	1.37 (0.77–2.43)	
Never married	212	13/358.0	3.6	1.63 (0.90–2.96)	
Education					
None	102	8/172.3	4.6	1.00	1.00
Primary	1426	67/2359.2	2.8	0.61 (0.29–1.27)	0.64 (0.30–1.34)
Secondary or more	846	21/1393.0	1.5	0.32 (0.14–0.73)∗∗	0.32 (0.14–0.74)∗∗
Nonmarital sexual partners in last 12 months					
0	1835	64/3016.5	2.1	1.00	1.00
1	506	30/851.7	3.5	1.66 (1.07–2.56)∗∗	1.44 (0.89–2.33)
2+	33	2/56.4	3.5	1.67 (0.41–6.83)	1.28 (0.30–5.41)
Social-economic status					
High	1311	50/2200.8	2.3	1.00	
Middle	722	28/1165.4	2.4	1.06 (0.66–1.68)	
Low	341	18/558.4	3.2	1.42 (0.83–2.43)	
Nugent score					
Normal	1093	42/1795.4	2.3	1.00	1.00
Intermediate	339	6/544.2	1.1	0.47 (0.20–1.11)	0.46 (0.20–1.09)
BV	942	48/1585.0	3.0	1.29 (0.86–1.96)	1.13 (0.74–1.72)
HIV status					
Negative	2069	69/3387.4	2.0	1.00	1.00
Positive	305	27/537	5.0	2.47 (1.58–3.85)∗∗	2.34 (1.44–3.78)∗∗
Syphilis serology					
Negative	2300	92/3802.6	2.4	1.00	1.00
Positive	74	4/122.0	3.3	1.36 (0.50–3.69)	1.09 (0.39–3.01)

^*^ *P* < 0.05. ∗∗*P* < 0.001.

**(b):** 

	*N*	Incident *T. vaginalis n*/PY	Incidence/100 py	Unadj.IRR 95% CI	Adj.IRR 95% CI
Overall	2374	96/3924.6	2.4		
HC use in last 12 months					
No HC/no condom	1054	45/1733.2	2.60	1.00	1.00
DMPA only	664	15/1089.9	1.4	0.53 (0.30–0.95)∗	0.54 (0.30–0.98)∗
COC only	133	5/220.3	2.3	0.87 (0.35–2.20)	1.02 (0.40–2.59)
Implants only	32	4/56.0	7.1	2.75 (1.00–7.65)∗	3.01 (1.07–8.49)∗
Condoms only	491	27/825.2	3.3	1.2 (0.78–2.03)	0.97 (0.57–1.64)
Age					
<20	165	11/277.7	4.0	1.00	1.00
20–29	939	40/1566.3	2.6	0.64 (0.33–1.26)	0.70 (0.34–1.43)
30–39	951	37/1565.8	2.4	0.60 (0.30–1.17)∗	0.57 (0.30–1.16)
40+	319	8/514.8	1.6	0.39 (0.16–0.98)∗∗	0.28 (0.11–0.73)∗∗
Education					
None	102	8/172.3	4.6	1.00	1.00
Primary	1426	67/2359.2	2.8	0.610 (0.29–1.27)	0.63 (0.30–1.32)
Secondary or more	846	21/1393.0	1.5	0.32 (0.14–0.73)∗∗	0.32 (0.14–0.74)∗∗
Nonmarital sexual partners in last 12 months					
0	1835	64/3016.5	2.1	1.00	1.00
1	506	30/851.7	3.5	1.66 (1.07–2.56)	1.49 (0.92–2.42)
2+	33	2/56.4	3.5	1.67 (0.41–6.83)	1.26 (0.30–5.28)
Social-economic status					
High	1311	50/2200.8	2.3	1.00	
Middle	722	28/1165.4	2.4	1.06 (0.66–1.68)	
Low	341	18/558.4	3.2	1.41 (0.83–2.43)	
Nugent score					
Normal	1093	42/1795.4	2.3	1.00	1.00
Intermediate	339	6/544.2	1.1	0.47 (0.20–1.11)	0.44 (0.19–1.05)
BV	942	48/1585.0	3.0	1.29 (0.86–1.96)	1.10 (0.72–1.69)
HIV status					
Negative	2069	69/3387.4	2.0	1.00	1.00
Positive	305	27/537	5.0	2.40 (1.42–4.08)∗∗	2.38 (1.47–3.86)∗∗
Syphilis serology					
Negative	2300	92/3802.6	2.4	1.00	1.00
Positive	74	4/122.0	3.3	1.36 (0.50–3.69)	1.16 (0.42–3.20)

^*^ *P* < 0.05. ∗∗*P* < 0.001.

**(c):** 

	*N*	Incident *T. vaginalis n*/PY	Incidence/100 py	Unadj.IRR 95% CI	Adj.IRR 95% CI
Overall	2374	96/3924.6	2.4		
HC use at baseline and follow-up					
Consistently used no method	653	30/1071.7	2.8	1.00	1.00
Consistently used HC	483	15/789.1	1.9	0.68 (0.36–1.26)	0.73 (0.39–1.40)
Initiated HC use at follow-up	244	9/402.7	2.2	0.80 (0.38–1.68)	0.76 (0.36–1.69)
Discontinued HC use at follow-up	215	3/350.6	0.86	0.30 (0.09–1.00)∗	0.30 (0.09–0.99)∗
Consistently used condoms	217	12/365.9	3.2	1.17 (0.60–2.29)	0.87 (0.42–1.79)
Others	562	27/944.5	2.8	1.02 (0.61–1.71)	0.81 (0.47–1.40)
Age					
<20	165	11/277.7	4.0	1.00	1.00
20–29	939	40/1566.3	2.6	0.64 (0.33–1.26)	0.71 (0.35–1.44)
30–39	951	37/1565.8	2.4	0.60 (0.30–1.17)	0.58 (0.28–1.17)
40+	319	8/514.8	1.6	0.39 (0.16–0.98)∗∗	0.28 (0.11–0.72)∗∗
Education					
None	102	8/172.3	4.6	1.00	1.00
Primary	1426	67/2359.2	2.8	0.610 (0.29–1.27)	0.68 (0.32–1.43)
Secondary or more	846	21/1393.0	1.5	0.32 (0.14–0.73)∗	0.34 (0.14–0.78)∗
Nonmarital sexual partners in last 12 months					
0	1835	64/3016.5	2.1	1.00	1.00
1	506	30/851.7	3.5	1.66 (1.07–2.56)	1.47 (0.92–2.36)
2+	33	2/56.4	3.5	1.67 (0.41–6.83)	1.29 (0.31–5.37)
Social-economic status					
High	1311	50/2200.8	2.3	1.00	
Middle	722	28/1165.4	2.4	1.06 (0.66–1.68)	
Low	341	18/558.4	3.2	1.41 (0.83–2.43)	
Nugent score					
Normal	1093	42/1795.4	2.3	1.00	1.00
Intermediate	339	6/544.2	1.1	0.47 (0.20–1.11)	0.46 (0.19–1.08)
BV	942	48/1585.0	3.0	1.29 (0.86–1.96)	1.14 (0.74–1.73)
HIV Status					
Negative	2069	69/3387.4	2.0	1.00	1.00
Positive	305	27/537	5.0	2.40 (1.42–4.08)∗∗	2.36 (1.44–3.85)∗∗
Syphilis serology					
Negative	2300	92/3802.6	2.4	1.00	1.00
Positive	74	4/122.0	3.3	1.36 (0.50–3.69)	1.07 (0.39–2.94)

^*^ *P* < 0.05. ∗∗*P* < 0.001.

**(d):** 

	*N*	Incident *T. vaginalis n*/PY	Incidence/100 py	Unadj.IRR 95% CI	Adj.IRR 95% CI
Overall	2374	96/3924.6	2.4		
HC use at baseline and follow-up					
Consistently used no method	653	30/1071.7	2.8	1.00	1.00
Consistently used DMPA	359	9/582.9	1.5	0.55 (0.26–1.16)	0.59 (0.28–1.26)
Consistently used COCs	50	2/84.2	2.4	0.85 (0.20–3.55)	1.07 (0.25–4.56)
Consistently used implants	28	4/49.8	8.0	2.87 (1.01–8.14)∗	3.13 (1.08–9.07)∗
Other categories	1284	51/2135.8	2.4	0.85 (0.54–1.34)	0.70 (0.44–1.13)
Age					
<20	165	11/277.7	4.0	1.00	1.00
20–29	939	40/1566.3	2.6	0.64 (0.33–1.26)	0.65 (0.32–1.31)
30–39	951	37/1565.8	2.4	0.60 (0.30–1.17)	0.52 (0.26–1.06)
40+	319	8/514.8	1.6	0.39 (0.16–0.98)∗∗	0.26 (0.10–0.67)∗∗
Education					
None	102	8/172.3	4.6	1.00	1.00
Primary	1426	67/2359.2	2.8	0.610 (0.29–1.27)	0.64 (0.30–1.38)
Secondary or more	846	21/1393.0	1.5	0.32 (0.14–0.73)	0.34 (0.14–0.78)∗
Nonmarital sexual partners in last 12 months					
0	1835	64/3016.5	2.1	1.00	1.00
1	506	30/851.7	3.5	1.66 (1.07–2.56)	1.61 (1.01–2.55)∗
2+	33	2/56.4	3.5	1.67 (0.41–6.83)	1.40 (0.33–5.77)
Social-economic status					
High	1311	50/2200.8	2.3	1.00	
Middle	722	28/1165.4	2.4	1.06 (0.66–1.68)	
Low	341	18/558.4	3.2	1.41 (0.83–2.43)	
Nugent score					
Normal	1093	42/1795.4	2.3	1.00	1.00
Intermediate	339	6/544.2	1.1	0.47 (0.20–1.11)	0.45 (0.19–1.06)
BV	942	48/1585.0	3.0	1.29 (0.86–1.96)	1.09 (0.71–1.66)
HIV status					
Negative	2069	69/3387.4	2.0	1.00	1.00
Positive	305	27/537	5.0	2.40 (1.42–4.08)	2.52 (1.56–4.08)∗∗
Syphilis serology					
Negative	2300	92/3802.6	2.4	1.00	1.00
Positive	74	4/122.0	3.3	1.36 (0.50–3.69)	1.07 (0.39–2.95)

^*^ *P* < 0.05. ∗∗*P* < 0.001.
